# Synthesis of Boronate Affinity-Based Oriented Dummy Template-Imprinted Magnetic Nanomaterials for Rapid and Efficient Solid-Phase Extraction of Ellagic Acid from Food

**DOI:** 10.3390/molecules29112500

**Published:** 2024-05-25

**Authors:** Daojin Li, Na Tang, Xiping Tian

**Affiliations:** Henan Key Laboratory of Fuction-Oriented Porous Materials, College of Chemistry and Chemical Engineering, Luoyang Normal University, Luoyang 471934, China; tangna0496@163.com (N.T.); tianxiping2002@163.com (X.T.)

**Keywords:** oriented surface imprinting, boronic acid, magnetic nanoparticles, dummy template, ellagic acid

## Abstract

Ellagic acid (EA) is a natural polyphenol and possesses excellent in vivo bioactivity and antioxidant behaviors, which play an important role in the treatment of oxidative stress-related diseases, such as cancer. Additionally, EA is also known as a skin-whitening ingredient. The content of EA would determine its efficacy. Therefore, the accurate analysis of EA content can provide more information for the scientific consumption of EA-rich foods and cosmetics. Nevertheless, the analysis of EA in these samples is challenging due to the low concentration level and the presence of interfering components with high abundance. Molecularly imprinted polymers are highly efficient pretreatment materials in achieving specific recognition of target molecules. However, the traditional template molecule (EA) could not be absolutely removed. Hence, template leakage continues to occur during the sample preparation process, leading to a lack of accuracy in the quantification of EA in actual samples, particularly for trace analytes. In addition, another drawback of EA as an imprinting template is that EA possesses poor solubility and a high price. Gallic acid (GA), called dummy templates, was employed for the synthesis of MIPs as a solution to these challenges. The approach used in this study was boronate affinity-based oriented surface imprinting. The prepared dummy-imprinted nanoparticles exhibited several significant advantages, such as good specificity, high binding affinity ((4.89 ± 0.46) × 10^−5^ M), high binding capacity (6.56 ± 0.35 mg/g), fast kinetics (6 min), and low binding pH (pH 5.0) toward EA. The reproducibility of the dummy-imprinted nanoparticles was satisfactory. The dummy-imprinted nanoparticles could still be reused even after six adsorption–desorption cycles. In addition, the recoveries of the proposed method for EA at three spiked levels of analysis in strawberry and pineapple were 91.0–106.8% and 93.8–104.0%, respectively, which indicated the successful application to real samples.

## 1. Introduction

**Figure 1:**
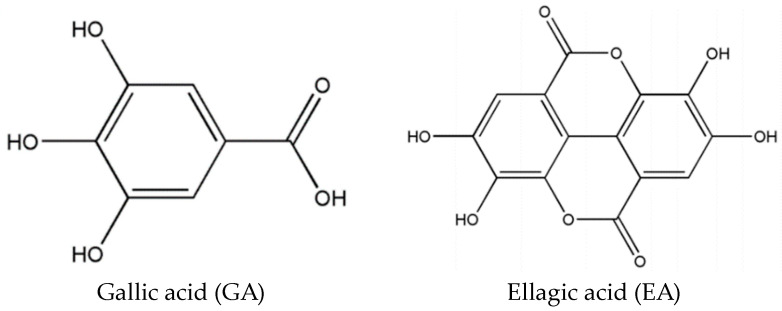
The molecular structures of gallic acid (GA) and ellagic acid (EA).

Polyphenols are widely distributed in organisms from plants to animals. The importance of polyphenols results from their important biological activities, as well as intriguing physical and chemical properties. Ellagic acid (EA) is a natural polyphenol that is widely found in various teas, nuts, fruits, and other plant tissues [1,2]. It is a dimeric derivative of gallic acid (GA). Figure 1 shows the molecular structures of GA and EA. EA exhibits outstanding in vivo bioactivity and antioxidant properties, making it a key player in the prevention of cancer [3,4,5], inflammation [6,7], and degenerative diseases [8,9]. In addition, it also demonstrates various beneficial pharmacological properties, including anti-inflammatory, anti-bacterial, anti-viral, and anti-obesity properties. EA functions as a food additive to mitigate food deterioration. Furthermore, in the cosmetics industry, EA is one of the most popular ingredients in many kinds of cosmetic products because of its satisfactory skin-whitening effect through inhibiting tyrosinase activity [10]. The concentration limit of EA in commercial cosmetics is 0.5% (*w*/*w*) in Taiwan [11]. As a result, EA has gained extensive usage in clinical research, cosmetics, and the food industry [12]. Clearly, the content of EA determines its efficacy. Therefore, the accurate quantification of EA content can provide more information for the scientific consumption of EA-rich foods and cosmetics. The literature suggests that the most commonly employed techniques for quantifying EA concentration in actual samples are a liquid chromatograph–mass spectrometer/mass spectrometer (LC-MS/MS) [13], high-performance liquid chromatography (HPLC) [14,15], capillary electrophoresis [16,17], and localized surface plasmon resonance [18]. Although LC-MS/MS possesses many advantages, especially good sensitivity and accuracy, it suffers several drawbacks, such as costly instruments, complicated sample preparation, and long time consumption. HPLC and capillary electrophoresis are preferred techniques for separating and quantifying polyphenols. HPLC is the most commonly used for determining EA. However, the method still requires sample pretreatment processes because of the limited presence of EA in real samples and the effect of interfering components.

Solid-phase extraction (SPE) is commonly employed for sample preparation in the analysis of phenolic compounds before HPLC [19]. Typically, commercial cartridges with C8 and C18 columns are utilized to extract the polyphenols from the samples [20]. However, the routine SPE suffered from the disadvantages of low selectivity, poor recovery, and complicated operation. Hence, there is a high demand for selective enrichment materials for pretreating EA samples.

Molecularly imprinted polymers (MIPs) are a novel type of adsorbent material that possess a unique three-dimensional cavity capable of specifically recognizing and interacting with target substances [21,22,23,24,25]. MIPs have multiple advantages compared with conventional absorbents, such as high specificity, easy preparation, and reduced solvent usage. Molecularly imprinted solid-phase extraction has been shown to be an efficient method for selectively separating and concentrating targets from complex matrices thanks to its favorable properties. However, conventional MIPs also faced several drawbacks. The traditional template molecule (EA) could not be absolutely removed. Hence, template leakage would continue to occur during the sample preparation process, leading to a lack of accuracy in the quantification of EA in actual samples, particularly for trace analytes [26,27]. Furthermore, employing the target analytes themselves as molecular templates for synthesizing MIPs would require substantial quantities of standard products. To address these issues, researchers utilized structural analogs of the analytes, referred to as dummy templates, to synthesize MIPs. These obtained MIPs displayed similar recognition specificity for the analytes [28,29]. So far, about five references on EA-imprinted polymers have been reported [30,31,32,33,34]. Therefore, there are only three references aiming at the fabrication of boronate affinity-based MIPs for the determination of EA in foodstuffs [31,32,34]. To our knowledge, there have been no reports about the preparation of dummy template-imprinted polymers for the determination of EA. It is widely recognized that EA has low solubility and high cost. Because EA is a dimeric derivative of GA, the molecular structure of GA is similar to that of EA, while GA has low cost and relatively good solubility. Hence, we employ GA as a dummy template to develop dummy template-imprinted polymers for the enrichment and detection of EA residue.

The conventional imprinting approach is the bulk imprinting method [21], which was widely used in the preparation of MIPs due to its simple operation. However, it has several drawbacks, including long time consumption, difficult template removal, a small binding capacity, and slow mass transfer. Fortunately, GA contains the structure of a cis-diol. Boronic acids are important functional monomers in imprinting cis-diol-containing compounds in covalent imprinting [21]. Until now, the strategy of boronate affinity-based oriented surface imprinting has been used to prepare a series of boronate affinity-based MIPs for targets [35,36,37,38,39,40]. The oriented surface imprinting approach can provide high binding capacity, easy template removal, and fast mass transfer. Magnetic Fe_3_O_4_ nanoparticles (MNPs) [41,42,43], due to their low cost, magnetic susceptibility, low toxicity, and good biocompatibility, can be used as supporting materials. In the past few years, various types of functionalized MNPs such as imprinted MNPs, metal-chelated MNPs, and other functional MNPs have been widely used in diverse fields such as solid-phase extraction, biomedicine, environmental applications, and wastewater treatment [41,44,45,46,47,48]. Therefore, boronic acid-functionalized MNPs can effectively serve as a solid substrate for immobilizing and eliminating GA. Moreover, the imprinting coating plays a crucial role in determining the binding properties of imprinted materials. The organic polymer has the potential to serve as an effective imprinting coating.

**Figure 2:**
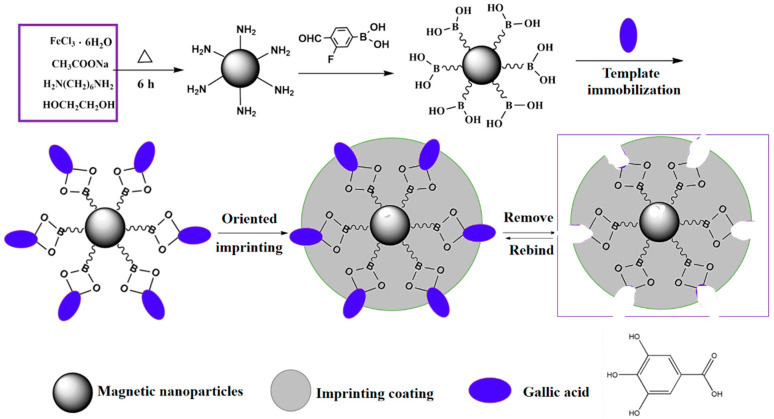
The process diagram of creating gallic acid (GA)-imprinted magnetic nanoparticles through boronate affinity-based oriented surface imprinting.

In this work, we attempt to prepare novel dummy GA-imprinted MNPs for the detection of EA residue in real samples for the first time. According to Figure 2, the imprinting process using a boronate affinity-based oriented surface imprinting approach can be divided into three steps. Initially, GA was attached to 3-fluoro-4-formylphenylboronic acid (FFPBA)-functionalized MNPs via boronate affinity interaction. Then, 2-anilinoethanol was self-polymerized on the MNP surface to form an imprinting coating with appropriate thickness. In general, the imprinting coating’s thickness should be adjusted to one-third to two-thirds of the molecular size of the template in any of the three dimensions. Finally, the GA template was removed by an acidic solution with sodium dodecyl sulfate (SDS) to form MIPs with an imprinting cavity containing FFPBA. Therefore, the 3D cavities created through imprinting conformed to both the size and shape of the template molecule. Because the obtained imprinting coating could cover excessive binding sites, non-specific adsorption can be effectively eliminated. The developed dummy-imprinted nanoparticles exhibited several significant advantages, such as good specificity, high binding affinity (4.89 ± 0.46) × 10^−5^ M), high binding capacity (6.56 ± 0.35 mg/g), fast kinetics (6 min), and low binding pH (pH 5.0) toward EA. The reproducibility was satisfactory. The dummy-imprinted nanoparticles could still be re-used, even after six adsorption–desorption cycles. Eventually, the developed dummy-imprinted nanoparticles were successfully applied to the analysis of EA in biological fluid samples.

## 2. Results and Discussion

### 2.1. Characterization of Fe_3_O_4_@FFPBA@MIPs

**Figure 3:**
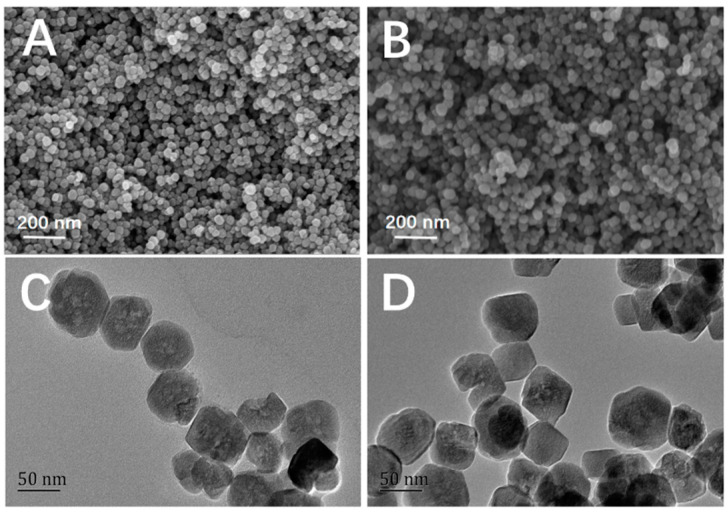
SEM images of bare Fe_3_O_4_ (**A**) and Fe_3_O_4_@FFPBA@MIPs (**B**); TEM images of bare Fe_3_O_4_ (**C**) and Fe_3_O_4_@FFPBA@MIPs (**D**).

The scanning electron microscopy (SEM) and transmission electron microscopy (TEM) images of bare Fe_3_O_4_ and Fe_3_O_4_@FFPBA@MIPs are shown in Figure 3A–D, respectively. It is evident in the figures that Fe_3_O_4_@FFPBA@MIPs have a nearly spherical shape and a relatively narrow size distribution, with an average diameter of approximately 50 nm. The result indicated that Fe_3_O_4_@FFPBA@MIPs had satisfactory dispersibility, which is highly advantageous for the selective recognition of EA. According to Figure 3BD, it can be found that the imprinting coating of the Fe_3_O_4_ nanoparticles was too thin to be clearly observed via SEM and TEM. For boronate affinity-based oriented surface imprinting, the thickness of the imprinting coating must be smaller than the molecular size of the template in one of the three dimensions. The length, width, or height of the GA molecule was less than 1 nm. Thus, the appropriate thickness of the imprinting layer must be less than 1 nm. Clearly, the very thin imprinting coating cannot be clearly observed, regardless of SEM and TEM.

**Figure 4:**
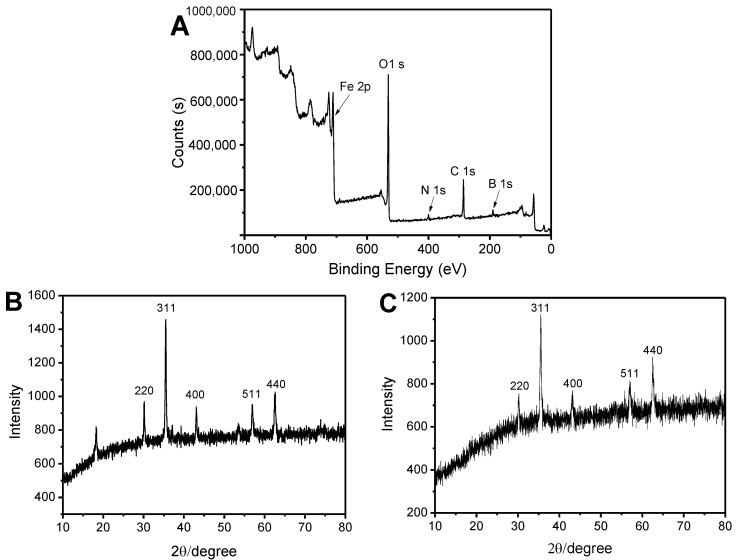
XPS spectra (**A**) of Fe_3_O_4_@FFPBA@MIP and XRD spectra of bare Fe_3_O_4_ (**B**) and Fe_3_O_4_@FFPBA@MIP (**C**).

In order to verify the successful preparation of Fe_3_O_4_@FFPBA@MIPs, X-ray photoelectron survey spectrometry (XPS) of Fe_3_O_4_@FFPBA@MIPs was investigated. As depicted in Figure 4A, the XPS peaks were observed at 286 eV for C 1s, 531 eV for O 1s, 711 eV for Fe 2p, 399 eV for N 1s, and 190 eV for B 1s. The peak at a binding energy of 190 eV indicates the presence of B atoms in Fe_3_O_4_@FFPBA@MIPs, confirming the successful preparation of Fe_3_O_4_@FFPBA@MIPs. Furthermore, the XRD analysis shown in Figure 4C confirmed the crystalline nature of Fe_3_O_4_@FFPBA@MIPs. In the curve of Fe_3_O_4_@FFPBA@MIPs, there are five clearly distinguishable strong diffraction peaks (2θ = 30.3°, 35.5°, 43.3 °, 53.4°, 57.0°, and 62.5°) observed. The peak positions are indexed as (220), (311), (400), (511), and (440) at the corresponding 2θ values. The data closely match the crystalline planes of the bare Fe_3_O_4_ nanoparticles’ cubic spinel nanostructure (Figure 4B). This result indicated that the structure of the carrier Fe_3_O_4_ was not changed during the coating process of the imprinted layer. In addition, the intensity of XRD peaks of Fe_3_O_4_@FFPBA@MIPs was weakened compared to that of bare Fe_3_O_4_ nanoparticles, which implied that Fe_3_O_4_@FFPBA@MIPs had an imprinted layer with a certain thickness.

### 2.2. Selectivity of Fe_3_O_4_@FFPBA

**Figure 5:**
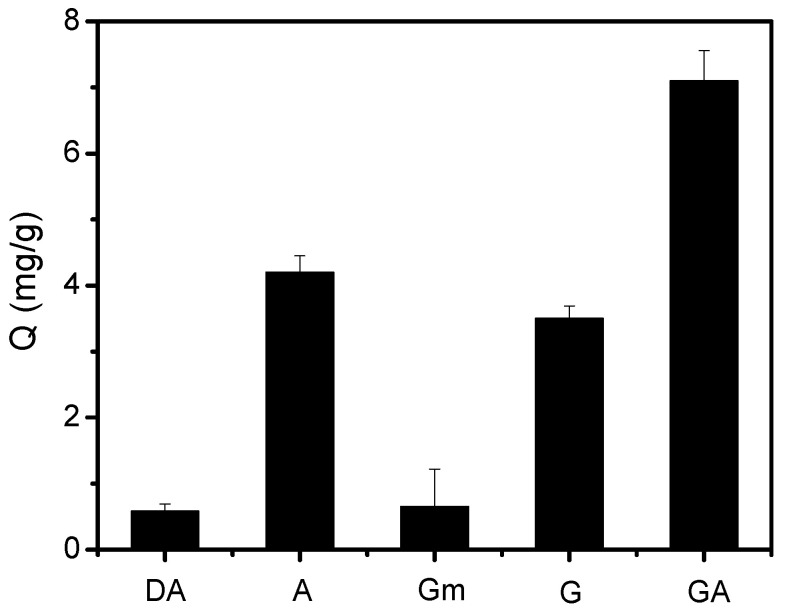
The binding capacity of Fe_3_O_4_@FFPBA for various analytes. The binding buffer used was 50 mM PBS (pH 7.0), while the elution solution consisted of 100 mM HAc (pH 2.7). The samples examined were DA, A, Gm, G, and GA dissolved in a binding buffer at a concentration of 1 mg/mL.

The post-modification of the carrier Fe_3_O_4_ with boronic acid is key for boronate affinity-based oriented surface imprinting. The post-modification of boronic acids can be confirmed by investigating the selectivity of the FFPBA-functionalized Fe_3_O_4_ for cis-diol-containing compounds. A and G were examined as compounds containing cis-diols, whereas DA and Gm were investigated as analogs lacking cis-diols. In Figure 5, the Fe_3_O_4_@FFPBA exhibits a higher binding amount for A or G than DA or Gm under neutral pH conditions (pH 7.0). It is clear that the boronic acid-modified MNPs demonstrated remarkable selectivity for cis-diol-containing compounds. Because EA contains two cis-diols, the Fe_3_O_4_@FFPBA exhibited a relatively high binding capacity for EA. Apparently, the MNPs functionalized with boronic acid demonstrated a strong preference for cis-diol-containing compounds. The successful immobilization of boronic acid FFPBA onto the Fe_3_O_4_ was confirmed by these results.

### 2.3. Investigation of Imprinting Conditions

For the boronate affinity-based oriented surface imprinting, the thickness of the imprinting coating on the surface of the Fe_3_O_4_@FFPBA substrate is key for the binding properties of Fe_3_O_4_@FFPBA@MIPs. The surface of Fe_3_O_4_@FFPBA was coated with a self-polymerized imprinting layer prepared from 2-anilinoethanol. 2-anilinoethanol was used as an imprinting monomer mainly because its hydrophilicity could eliminate non-specific adsorption and its self-polymerization could be well controlled on imprinting coating by concentration and time.

**Figure 6:**
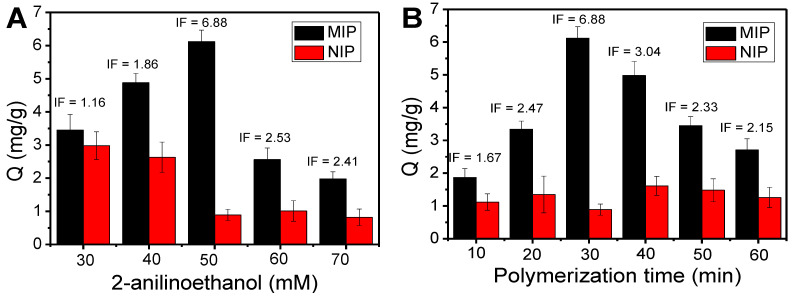
Effects of imprinting conditions on the target amount of ellagic acid (EA) captured by Fe_3_O_4_@FFPBA@MIP and the imprinting factor (IF). (**A**) Concentration of 2-anilinoethanol; (**B**) polymerization time.

Typically, the thickness of the imprinting coating needs to be smaller than that of the template. We are aware that the thickness of the imprinting coating is correlated with the concentration of 2-anilinoethanol and polymerization time. The impact of the two factors on the imprinting effect was systematically assessed by the use of IF. Because the thickness of the imprinting coating on Fe_3_O_4_@FFPBA is in direct proportion to the concentration of 2-anilinoethanol, the most appropriate concentration of 2-anilinoethanol could be evaluated by the binding capacity of MIP and NIP at different 2-anilinoethanol concentrations. Based on the data shown in Figure 6A, it was determined that the optimal concentration of 2-anilinoethanol was 50 mM, resulting in an IF value of 6.88. In addition, the influence of polymerization time on the imprinting effect was also investigated. As depicted in Figure 6B, the binding capacity of MIP for EA gradually increased as time goes on from 10 to 30 min. However, when the polymerization time exceeds 30 min, the binding capacity of MIP for EA decreases, while NIP remains almost constant. Clearly, the changing trends of the IF were in good accordance with that of the binding amount of MIP. Thus, the optimal imprinting conditions for EA was the self-polymerization of 50 mM 2-anilinoethanol for 30 min, which provided the best IF of 6.88. One possible explanation for this phenomenon is that there were no boronic acid moieties present outside of the imprinted cavities in the current imprinting method.

### 2.4. Specificity of Fe_3_O_4_@FFPBA@MIPs

**Figure 7:**
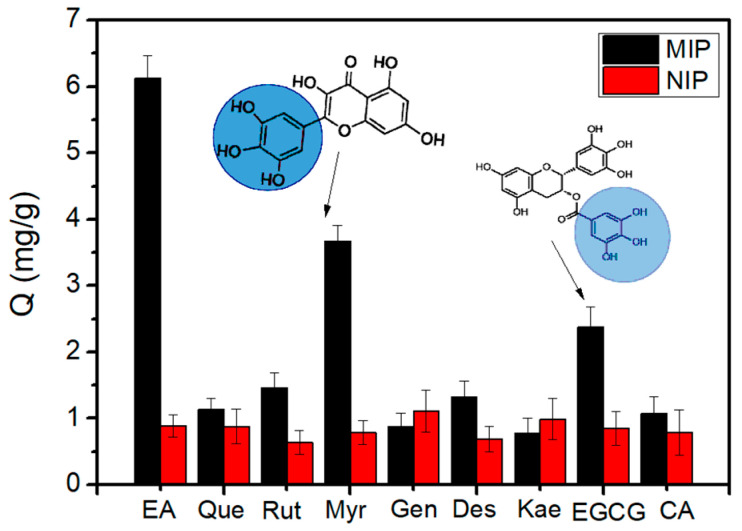
Comparison between the quantities of different compounds captured by Fe_3_O_4_@FFPBA@MIP and Fe_3_O_4_@FFPBA@NIP. Binding buffer: 50 mM PBS containing (pH 7.0); elution solution: 100 mM HAc (pH 2.7); samples: 0.5 mg/mL EA, Que, Rut, Myr, Gen, Des, Kae, EGCG, and CA dissolved in a binding buffer.

In order to evaluate the specificity of Fe_3_O_4_@FFPBA@MIPs for EA, several compounds, including Que, Rut, Myr, Gen, Des, and Kae, were selected as the competitive substances. Fe_3_O_4_@FFPBA@MIPs demonstrated a higher binding capacity for EA when compared to other competitive compounds, as illustrated in Figure 7. Although Que, Rut, and Myr contain cis-diols, Fe_3_O_4_@FFPBA@MIPs also exhibited a relatively low binding capacity for these compounds. This result implied that the boronate affinity-based oriented surface imprinting strategy gained great success. Fe_3_O_4_@FFPBA@MIPs showed exceptional specificity for EA. However, it should be noted that the binding capacity of EA was only a little higher than that of Myr or EGCG for Fe_3_O_4_@FFPBA@MIPs. This reason for the similar binding capacity is probably that the partial structure of Myr or EGCG is analogous to that of GA (see Figure 7).

### 2.5. Binding pH

**Figure 8:**
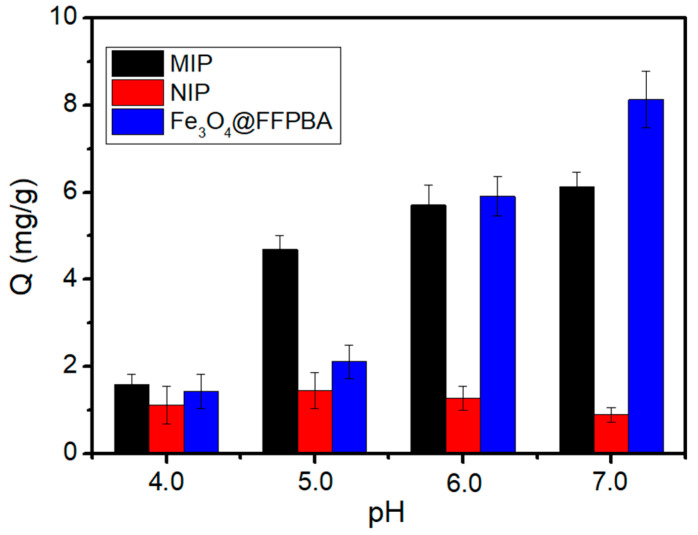
Ellagic acid (EA) binding capacity of Fe_3_O_4_@FFPBA@MIP, Fe_3_O_4_@FFPBA@NIP, and Fe_3_O_4_@FFPBA at different pH values. Sample: 1 mg/mL EA dissolved in a 50 mM phosphate buffer (pH 4.0, 5.0, 6.0, and 7.0).

The binding pH plays a crucial role in determining the binding property, and it has a positive correlation with the pKa value of boronic acid ligands. Additionally, the binding affinity of boronic acid ligands and supporting materials toward cis-diols also influenced the binding pH. The structures of the boronic acid ligands and supporting materials determine the binding affinity [21]. Due to the presence of electron-withdrawing groups, the FFPBA used exhibited a low p*K*_a_ value of 5.8 [49]. The boronic acid ligand was able to work at a relatively low pH condition of 6.0 (Figure 8). In addition, the imprinted cavities in Fe_3_O_4_@FFPBA@MIPs can provide higher binding affinity and thereby lead to lower binding pH values. To this end, we examined the influence of pH on the binding capability of Fe_3_O_4_@FFPBA@MIPs and Fe_3_O_4_@FFPBA@NIPs. As depicted in Figure 8, Fe_3_O_4_@FFPBA@MIPs exhibited a lower binding pH value (pH 5.0) compared to the Fe_3_O_4_@FFPBA (pH 6.0), whereas Fe_3_O_4_@FFPBA@NIPs showed minimal binding capacity for EA. The binding pH shift was due to the imprinted cavities in the structure of Fe_3_O_4_@FFPBA@MIPs. The cis-diols of EA are more prone to oxidation at basic pH, despite the fact that the binding affinity is higher at alkaline pH compared to neutral or acidic pH. Thus, such a lower binding pH for Fe_3_O_4_@FFPBA@MIPs is beneficial for the separation of EA from real samples.

### 2.6. Binding Equilibrium

**Figure 9:**
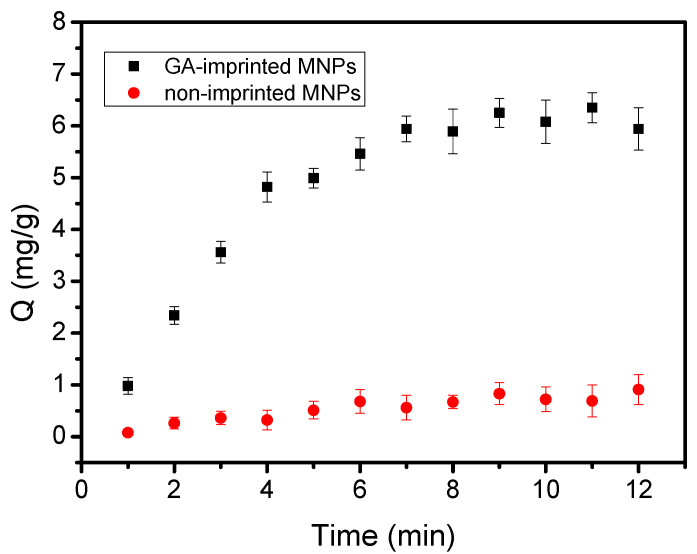
Binding equilibrium of Fe_3_O_4_@FFPBA@MIP and Fe_3_O_4_@FFPBA@NIP. Sample: 1.0 mg/mL ellagic acid (EA) containing 50 mM PBS, pH 7.0.

In order to evaluate the binding equilibrium time of Fe_3_O_4_@FFPBA@MIPs toward EA, the effect of response time on binding capacity (Q) was investigated (Figure 9). As seen in Figure 9, Fe_3_O_4_@FFPBA@MIPs had a faster adsorption rate than Fe_3_O_4_@FFPBA@NIPs within the first 4 min. The adsorption rate slowed down when the response time exceeded 4 min, and the binding reaction reached equilibrium at 6 min. This implied that the majority of the binding sites were already occupied by EA in this particular scenario. Clearly, the equilibrium time of Fe_3_O_4_@FFPBA@MIPs for EA was lower than that of other imprinted polymers (15–420 min) [50,51,52,53,54,55,56,57,58,59]. The quicker adsorption kinetics may result from the use of boronic acid ligands and the oriented surface imprinting method. This result indicated that Fe_3_O_4_@FFPBA@MIPs for EA showed good binding kinetics.

### 2.7. Determination of K_d_ and Q_max_

**Figure 10:**
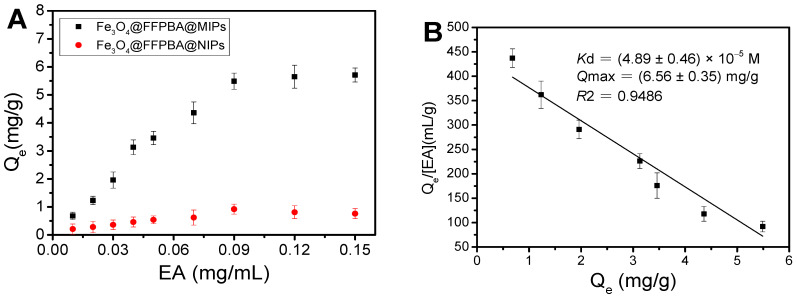
(**A**) Binding isotherms for binding of Fe_3_O_4_@FFPBA@MIPs and Fe_3_O_4_@FFPBA@NIPs to ellagic acid (EA), and (**B**) Scatchard plots for the binding of the Fe_3_O_4_@FFPBA@MIP to ellagic acid (EA).

The binding affinity of Fe_3_O_4_@FFPBA@MIPs can determine how low concentrations of EA can be extracted by Fe_3_O_4_@FFPBA@MIPs. In order to evaluate the binding affinity, the binding isotherm of Fe_3_O_4_@FFPBA@MIPs toward EA was investigated. As shown in Figure 10A, Fe_3_O_4_@FFPBA@MIPs demonstrated significantly greater binding capacity toward EA compared to Fe_3_O_4_@FFPBA@NIPs. According to the binding isotherm, the Scatchard plot (Figure 10B) for Fe_3_O_4_@FFPBA@MIPs was obtained to provide *Q*_max_ and *K*_d_ values of Fe_3_O_4_@FFPBA@MIPs. The calculated values for *Q*_max_ and *K*_d_ were (6.56 ± 0.35) mg/g and (4.89 ± 0.46) × 10^−5^ M, respectively. The strong binding strength of the prepared Fe_3_O_4_@FFPBA@MIPs for EA favors the extraction of EA with trace concentration.

### 2.8. Reproducibility and Reusability

**Figure 11:**
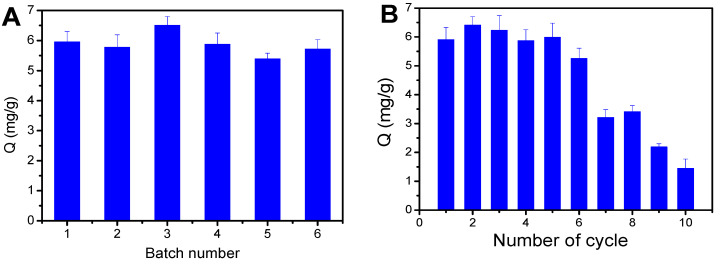
Batch-to-batch reproducibility of Fe_3_O_4_@FFPBA@MIP (**A**); reusability of Fe_3_O_4_@FFPBA@MIP (**B**). Sample: 1 mg/mL ellagic acid (EA) containing 50 mM PBS, pH 7.0.

The reproducibility of Fe_3_O_4_@FFPBA@MIPs was assessed by preparing six batches on different days and conducting three parallel measurements for each batch. As shown in Figure 11A, the six batches prepared using Fe_3_O_4_@FFPBA@MIPs exhibited similar binding capacities for EA. Furthermore, Fe_3_O_4_@FFPBA@MIPs exhibited relatively low standard deviations (RSDs) below 7.1% (specifically 5.7%, 4.5%, 6.3%, 3.5%, and 5.4%). The results suggested that the reproducibility of Fe_3_O_4_@FFPBA@MIPs was satisfactory due to the advantages of boronate affinity-based oriented surface imprinting.

Compared with native antibodies, one of the main advantages of MIPs is their ability to be reused. Thus, the reusability of Fe_3_O_4_@FFPBA@MIPs was investigated, and the adsorption–desorption cycle was repeated ten times using the same batch of Fe_3_O_4_@FFPBA@MIPs (Figure 11B). The adsorption capacity of Fe_3_O_4_@FFPBA@MIPs remained unchanged, even after undergoing six cycles of adsorption and desorption. It is evident that Fe_3_O_4_@FFPBA@MIPs are capable of being reused, even after undergoing six adsorption–desorption cycles. Thus, Fe_3_O_4_@FFPBA@MIPs possess high chemical stability.

### 2.9. Determination of EA in Real Samples

In order to investigate the performance of Fe_3_O_4_@FFPBA@MIPs in real samples, the selective separation and determination of EA from strawberry and pineapple samples by Fe_3_O_4_@FFPBA@MIP exaction was carried out. The obtained results are given in Table 1. Clearly, the content of EA in strawberry and pineapple samples was evaluated to be 12.6 and 1.6 μg/g, respectively. In addition, to investigate the accuracy of the method by selective separation and determination of EA in real samples, the evaluation of EA in EA-spiked strawberry and pineapple was performed. The recoveries were investigated with three standard amounts of EA added in strawberry and pineapple solutions, and the spiked concentration was fixed at 20, 40, and 60 μg/g, respectively. By extraction and determination of EA, the obtained recoveries for strawberry and pineapple solutions are shown in Table 1. Clearly, the recoveries of EA for strawberry and pineapple solutions were obtained to be 91.0–106.8% and 93.8–104.0%, respectively. In addition, the RSD values for strawberry and pineapple solutions were calculated to be ranging from 2.7–5.6 to 2.9–4.1%, respectively. The results showed that the proposed method is accurate, sensitive, and specific when determining the presence of EA in the strawberry and pineapple samples.

## 3. Experimental

### 3.1. Reagents and Materials

Adenosine (A), deoxyadenosine (DA), guanosine (G), 2′-O-methylguanosine (Gm), gallic acid (GA), ellagic acid (EA), quercetin (Que), rutin (Rut), myricetin (Myr), genistein (Gen), desonide (Des), kaempferol (Kae), epigallocatechin gallate (EGCG), chromogenic acid (CA), 2-anilinoethanol, ferric chloride hexahydrate (FeCl_3_·6H_2_O), 3-fluoro-4-formylphenylboronic acid (FFPBA), sodium dodecyl sulfate (SDS),anhydrous methanol, and anhydrous sodium acetate (NaOAc) are from J&K scientific (Shanghai, China). All other reagents were of at least analytical grade and used without further treatment.

### 3.2. Instruments

Scanning electron microscopy (SEM) analyses were performed on a Zeiss Sigma 500. Transmission electron microscopy (TEM) characterization was performed on a JEM-1010 system (JEOL, Tokyo, Japan). UV absorbance and the adsorption isotherm measurements were carried out with a U-3010 UV spectrophotometer equipped with a 1 cm cuvette (Kyoto, Japan). X-ray photoelectron spectroscopy (XPS) was performed with an ESCALAB 250Xi X-ray photoelectron spectrometer (Thermo, Waltham, MA, USA) with Al Kα radiation (hv = 1486.6 eV). The instrument was calibrated against the C1s band at 284.8 eV. Powder X-ray diffraction (XRD) analyses were carried out using a Bruker D8 Advance diffractometer with Cu Kα radiation, and the scanning angle ranged from 10° to 80° of 2θ. The HPLC profiles were recorded using a Hitachi HPLC system and a C18 column (5 µm, 4.6 mm × 150 mm). The column temperature was 30 °C.

### 3.3. Preparation of FFPBA-Functionalized MNPs (Fe_3_O_4_@FFPBA)

As depicted in Figure 1, Fe_3_O_4_@FFPBAs were prepared through the following two-step reactions. (1) Synthesis of Fe_3_O_4_ with NH_2_ and (2) functionalization of Fe_3_O_4_@FFPBA using FFPBA by the Schiff base reaction. The synthesis of Fe_3_O_4_ with NH_2_ in step (1) was conducted following a method previously described [31]. The next stage involved functionalizing Fe_3_O_4_ with FFPBA. A total of 300 mg of Fe_3_O_4_ with NH_2_ was added to 200 mL of anhydrous methanol that contained 2.0 g of FFPBA, and the resulting mixture was stirred for 12 h. Next, 2 g of sodium cyanoborohydride was added to the previous solution every 6 h for a total of 24 h. The Fe_3_O_4_@FFPBA was magnetically separated from the mixtures and subsequently cleansed with ethanol. The dried Fe_3_O_4_@FFPBA of 250 mg was obtained using vacuum drying (40 °C) and needed cold preservation.

### 3.4. Selectivity of the Fe_3_O_4_@FFPBA

The selectivity of the Fe_3_O_4_@FFPBA was assessed by testing A, G, and GA as cis-diol compounds, and DA and Gm as non-cis-diol analogs. The cis-diols and non-cis-diols solutions at concentrations of 1 mg/mL were obtained by dissolving them in a 50 mM phosphate buffer solution (PBS, pH 7.0). Each Fe_3_O_4_@FFPBA of 3 mg was separately dispersed into 1 mL of the above solution, and the resulting mixture was shaken at room temperature for 2 h. Then, the obtained target-immobilized Fe_3_O_4_@FFPBAs were collected by magnetic force and rinsed with 500 μL of PBS solution (pH 7.0) three times. The target-treated Fe_3_O_4_@FFPBAs were eluted with acetic acid solution for 1 h, and the obtained eluates containing targets were collected. The eluates that were obtained were assessed using UV absorbance at 260 nm. The measurement was conducted three times simultaneously.

### 3.5. Preparation of Fe_3_O_4_@FFPBA@MIPs

GA-imprinted Fe_3_O_4_@FFPBAs (Fe_3_O_4_@FFPBA@MIPs) were synthesized using a boronate affinity-based oriented surface imprinting method. As depicted in Figure 1, GA templates were first immobilized onto the Fe_3_O_4_@FFPBA. Specifically, 100 mg of the Fe_3_O_4_@FFPBA were dispersed into 20 mL PBS (pH 7. 0) containing GA, and, subsequently, the suspension was shaken at 25 °C for 1 h. The collected Fe_3_O_4_@FFPBA immobilized by the obtained GA was washed with PBS (pH 7.0) three times. Then, 80 mg of GA-immobilized Fe_3_O_4_@FFPBA were dispersed into 10 mL 2-anilinoethanol solution of 100 mM in pH 7.0 PBS and shaken at 25 °C for 5 min. Afterward, a 10 mL solution of 40 mM APS was added to the suspension obtained above. The above-obtained mixture was quickly sealed and agitated for 30 min at 25 °C. The self-polymerization of 2-anilinoethanol formed the imprinted polymer layer. The collected GA-imprinted Fe_3_O_4_@FFPBA was washed three times with water and ethanol. Afterward, GA templates were eliminated by 100 mM acetic acid and subsequently rinsed with water and ethanol, respectively. For the preparation of non-imprinted Fe_3_O_4_@FFPBAs (Fe_3_O_4_@FFPBA@NIPs), the operation is the same as the above, except there are no GA templates.

### 3.6. Optimization of Imprinting Conditions

The above-obtained Fe_3_O_4_@FFPBA@MIPs were prepared through the optimal imprinting conditions, which were obtained by studying the imprinting effect. To optimize the imprinting conditions, a GA solution of 1 mg/mL in 50 mM PBS (pH 7.0) was applied as a template solution. The imprinting conditions primarily consist of the concentration of 2-anilinoethanol and polymerization time. Each centrifuge tube was supplemented with the equivalent Fe_3_O_4_@FFPBA. Then, 500 μL of GA template solution was added into the above-mentioned centrifuge tube and shaken for 1 h at 25 °C. After washing the GA-immobilized Fe_3_O_4_@FFPBA with PBS with a pH 7.0 for 2 to 3 times, a specific amount of 2-anilinoethanol solution and APS solution were added to achieve concentrations of 30, 40, 50, 60, and 70 mM for 2-anilinoethanol. In addition, polymerization time was performed for 10–60 min. 

The imprinting factor (IF) was calculated by the ratio of *Q*_MIPs_ to *Q*_NIPs_ for EA, which was used to investigate the imprinting effect of Fe_3_O_4_@FFPBA@MIPs toward EA. The adsorption capacities of Fe_3_O_4_@FFPBA@MIPs and Fe_3_O_4_@FFPBA@NIPs for EA are represented by Q_MIPs_ and Q_NIPs_ (mg/g), respectively.

### 3.7. Specificity of Fe_3_O_4_@FFPBA@MIPs 

The specificity of Fe_3_O_4_@FFPBA@MIPs for EA was evaluated using seven samples, including EA, Que, Rut, Myr, Gen, Des, and Kae. First, the equivalent Fe_3_O_4_@FFPBA@MIPs were added to each centrifugal tube of 1.5 mL, respectively. Each sample was prepared separately with 50 mM PBS at pH 7.0 at a concentration of 1 mg/mL. Each sample solution of 500 μL was added into the above-mentioned centrifuge tube with equivalent Fe_3_O_4_@FFPBA@MIPs and shaken at 25 °C for 1 h. Fe_3_O_4_@FFPBA@MIPs were washed three times with 200 μL 50 mM PBS and then eluted in a 100 μL acetic acid solution (pH 2.7) for 2 h. The UV absorbance of the eluent was measured at 360 nm, 376 nm, 477 nm, 360 nm, 260 nm, 360 nm, and 365 nm for EA, Que, Rut, Myr, Gen, Des, and Kae, respectively. The measurement was repeated three times.

### 3.8. Binding Isotherm and Scatchard Analysis

The dissociation constant (*K*_d_) and maximum binding capacity (*Q*_max_) were determined according to a previously reported method [42,43]. A series of EA solutions with different concentrations dissolved in PBS (pH 7.0) were prepared in centrifuge tubes. We mixed equivalent Fe_3_O_4_@FFPBA@MIPs (3 mg each) with 500 μL of EA solutions at various concentrations (0.01, 0.02, 0.03, 0.04, 0.05, 0.07, 0.09, 0.12, and 0.15 mg/mL) in centrifuge tubes. The mixture solutions were then shaken on a rotator for 1 h at room temperature. Fe_3_O_4_@FFPBA@MIPs were collected, rinsed, and eluted with PBS (pH 7.0) and acetic acid solution, respectively. The eluates were used for measuring EA. The dissociation constant (*K*_d_) and apparent maximum binding capacity (*Q*_max_) were calculated based on the following Scatchard equation [42,43]:

$$\frac{Q_{e}}{C_{s}}=\frac{Q_{max}}{K_{d}}-\frac{Q_{e}}{K_{d}}$$

The binding capacity of Fe_3_O_4_@FFPBA@MIPs for EA at equilibrium is represented by *Q*_e_, while the free concentration at adsorption equilibrium is represented by *C*_s_. The slope and intercept of the *Q*_e_/*C*_s_ versus *Q*_e_ plots can be used to calculate the values of *K*_d_ and *Q*_max_.

### 3.9. Analysis of EA in Real Samples

Fresh strawberries and pineapples (10 g) were measured and blended with 40 mL of methanol. The resulting mixture was then heated for 2 h in a reflux apparatus. The refluxed sample was vacuum filtered after refluxing. The filtrate was evaporated, and the obtained samples were added to 100 mL of PBS pH 7.0 solution. In order to determine EA from the fruit solution, 15 mg of Fe_3_O_4_@FFPBA@MIPs was dispersed into 10 mL of the above sample solution and shaken for 10 min at room temperature. Fe_3_O_4_@FFPBA@MIPs that absorbed EA were eluted with acetic acid, and the eluent was collected and measured by UV absorbance. To assess the effectiveness of EA in fruit samples, various quantities of EA were added to the fruit samples to create strawberry or pineapple solutions with EA concentrations at different levels (20, 40, and 60 μg/g). Then, equivalent Fe_3_O_4_@FFPBA@MIPs were placed in the above-prepared 10 mL sample solution. Fe_3_O_4_@FFPBA@MIPs after adsorption were collected and washed with deionized water. Next, EA-adsorbed Fe_3_O_4_@FFPBA@MIPs were eluted with 2 mL acetic acid solution (pH 2.7) three times, and 6 mL of eluent was collected. The obtained eluent was analyzed by HPLC. The measurement was repeated three times.

## 4. Conclusions

In this study, we used boronate affinity-based template-immobilized surface imprinting to prepare dummy template-imprinted magnetic nanomaterials for rapid and efficient separation, enrichment, and purification of EA for the first time. The incorporation of boronic acid FFPBA in dummy template-imprinted magnetic nanomaterials exhibited several significant advantages, such as good specificity, high accuracy, high binding capacity, fast kinetics, and low binding pH toward EA. Furthermore, the adoption of a dummy template effectively avoided residual template leakage and ensured reliability. The prepared dummy template-imprinted magnetic nanomaterials were also applied for detecting EA in real strawberry and pineapple samples, and the experimental results revealed that imprinted magnetic nanomaterials were efficient SPE adsorbents for EA. This work provided a new strategy for the separation and enrichment of ellagic acid in food and cosmetics. In the future, we anticipate significant advancements and exciting opportunities for this approach.

## Figures and Tables

**Table 1:** Results of sample assay and recoveries for the determination of ellagic acid (EA) (n = 3).

Samples	Spiked Levels (μg/g)	Found (μg/g)	Recoveries (%)	RSD (%)
Strawberry	0.0	12.6	--	--
	20.0	30.8	91.0	4.3
	40.0	55.3	106.8	2.7
	60.0	69.8	95.3	5.6
Pineapple	0.0	1.6	--	--
	20.0	22.4	104.0	3.8
	40.0	39.1	93.8	2.9
	60.0	5.84	94.7	4.1

## Data Availability

The original contributions presented in the study are included in the article, further inquiries can be directed to the corresponding authors.
